# Bronchial Mucoepidermoid Carcinoma

**DOI:** 10.5334/jbr-btr.1215

**Published:** 2016-11-24

**Authors:** Christopher Gieraerts, Marijke Proesmans, Kate Sauer, Maria-Helena Smet, Luc Breysem

**Affiliations:** 1 UZ Leuven, BE

**Keywords:** mucoepidermoid carcinoma, endobronchial tumor, ct, pediatrics

## Case

**Figure 1:**
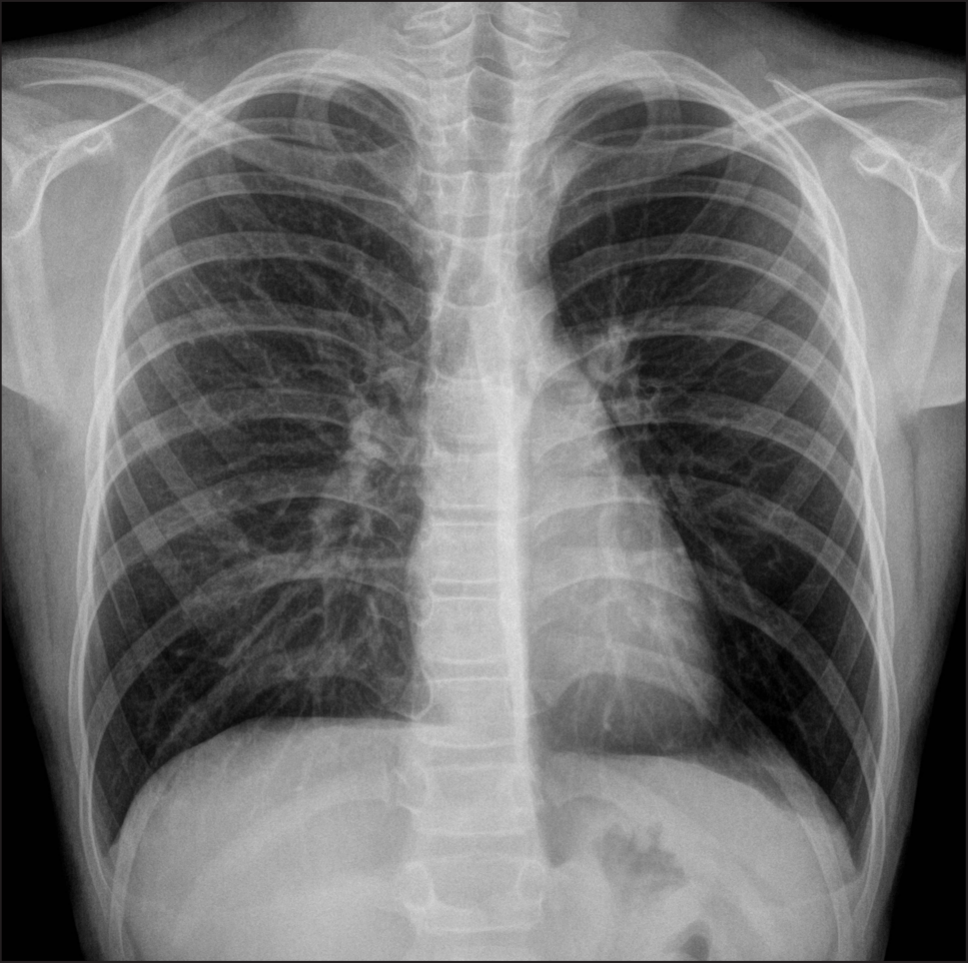
Standard chest radiograph.

**Figure 2:**
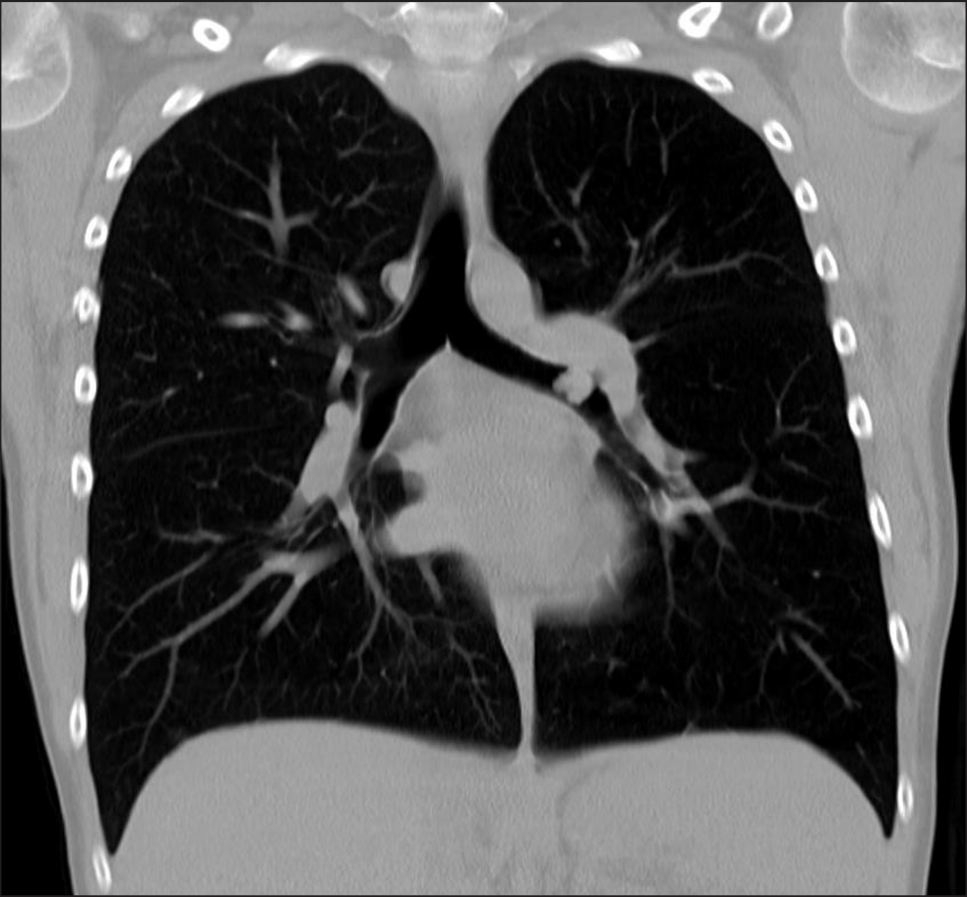
Coronal thick slice CT.

**Figure 3:**
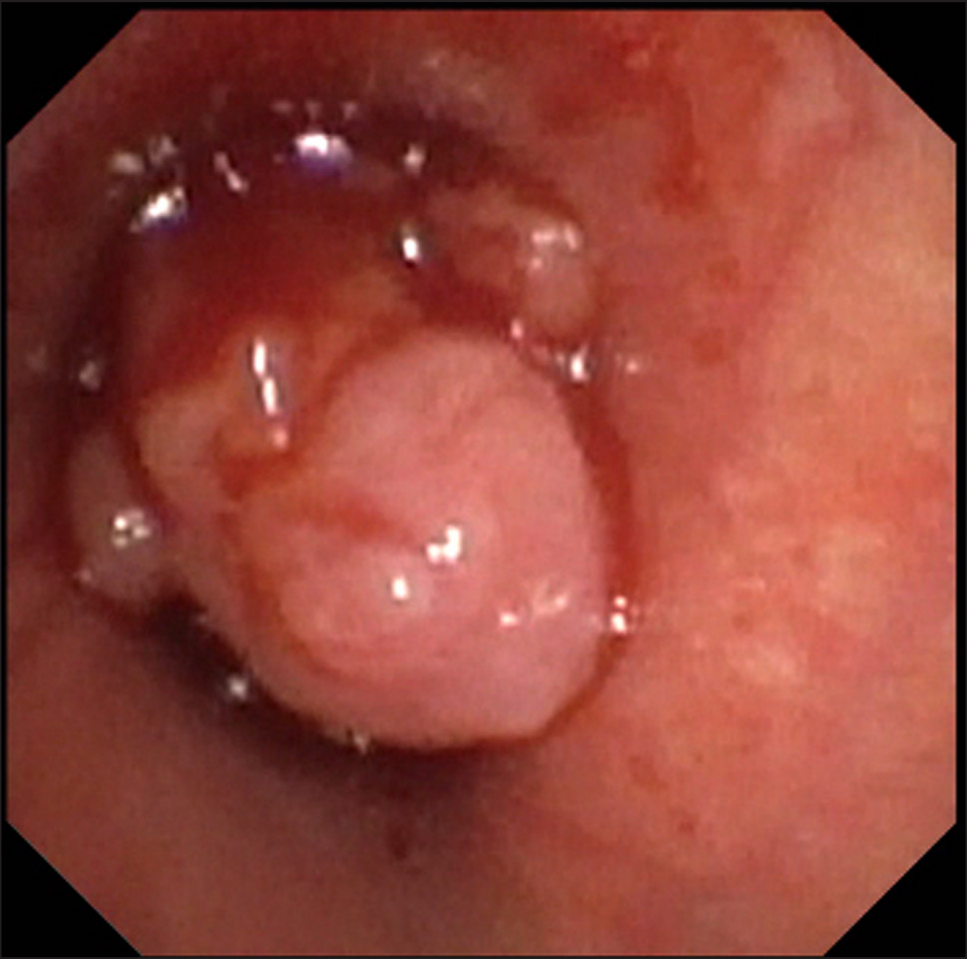
Bronchoscopic view in the left main bronchus.

A 12-year-old boy with chronic respiratory complaints, including cough, exercise intolerance, and persistent wheezing, was referred to our hospital because of persistent symptoms under extensive asthma therapy. A standard radiograph of the chest detected very slight hyperinflation of the left lung with increased lung translucency (Figure 1). Computed tomography revealed a polypoid mass distally in the left main bronchus with a density around 55 HU and very slightly decreased attenuation of the left lung, probably due to secondary air trapping (Figure 2). Bronchoscopy confirmed this finding, and biopsy revealed the mass to be a low-grade mucoepidermoid carcinoma (Figure 3). A curative bronchial sleeve resection was performed with partial mediastinal lymphadenectomy. All lymph nodes were normal, and there is no recurrence to this date (one year). Adjuvant chemotherapy was not indicated.

## Comment

Endobronchial tumors in children are very rare. The most frequent endobronchial lesions are carcinoid tumors and mucoepidermoid carcinomas 1. The latter are frequent tumors in the salivary glands but only rarely occur in the bronchi. Because of the typical endobronchial location, children typically present with recurrent wheezing and cough or recurrent pneumonia. Mucoepidermoid carcinomas arise in the submucosal bronchial glands and consist of mucus-secreting cells, squamous cells, and intermediate cells. The predominance of cell types acts as one of the grading criteria. Almost all mucoepidermoid carcinomas are low-grade tumors and require surgery but no adjuvant therapy. If resected completely, they have an excellent prognosis 1.
